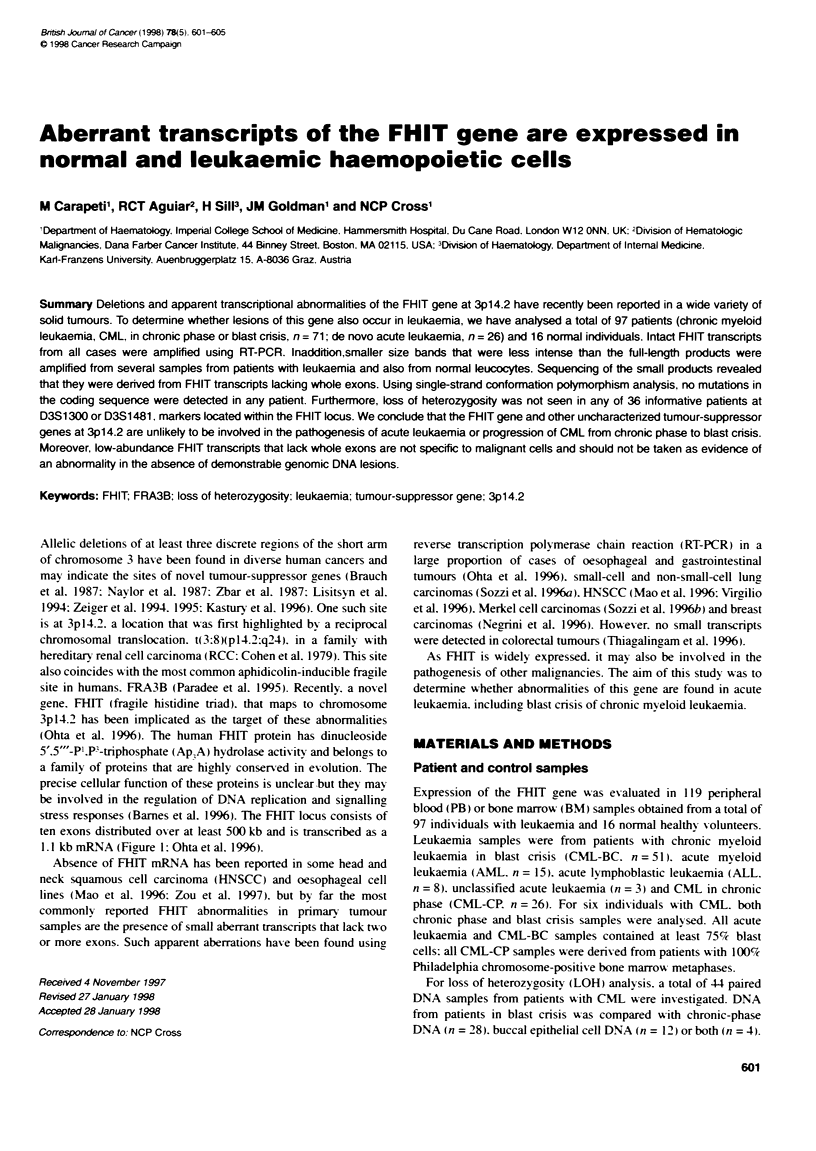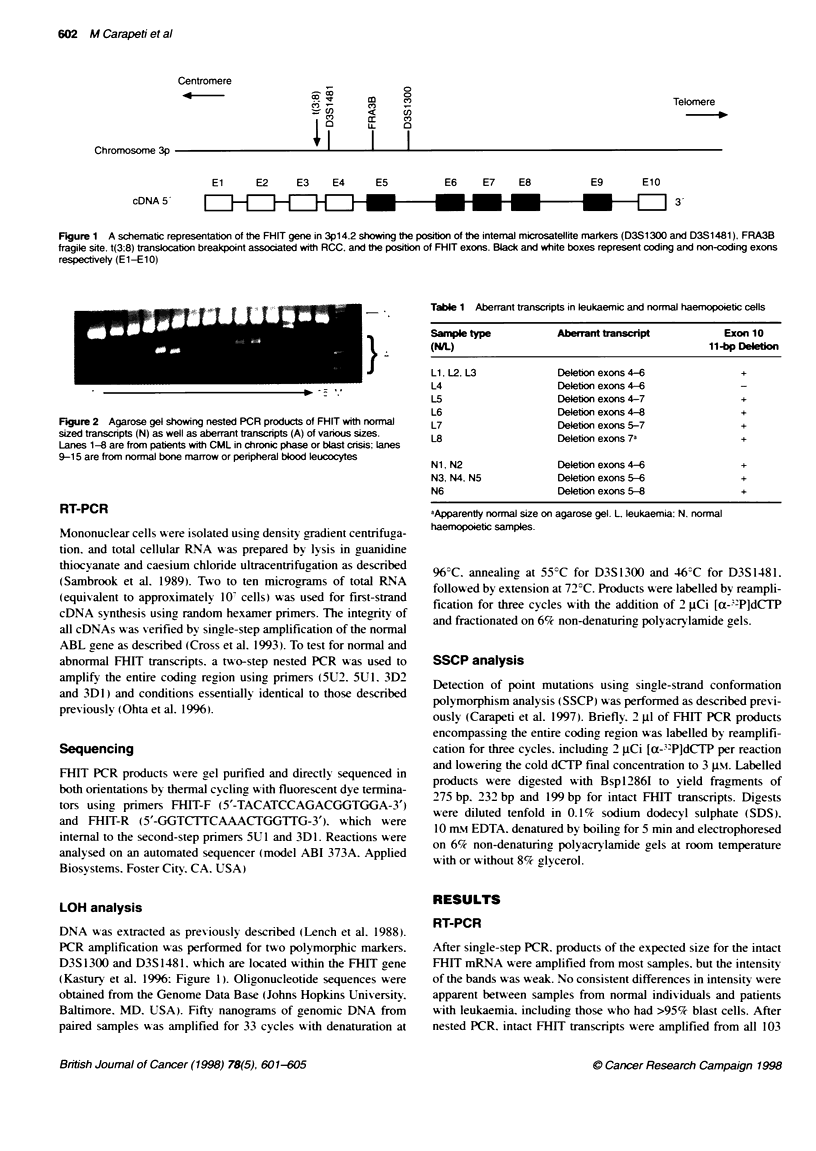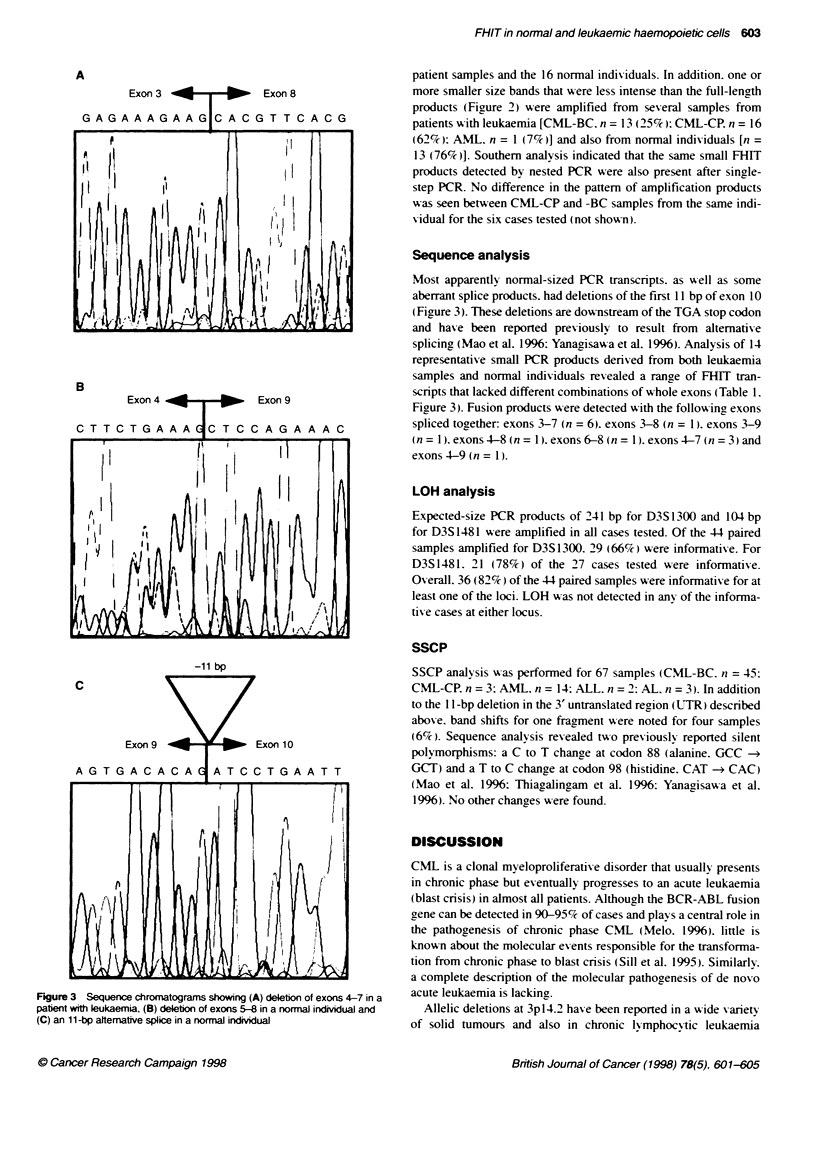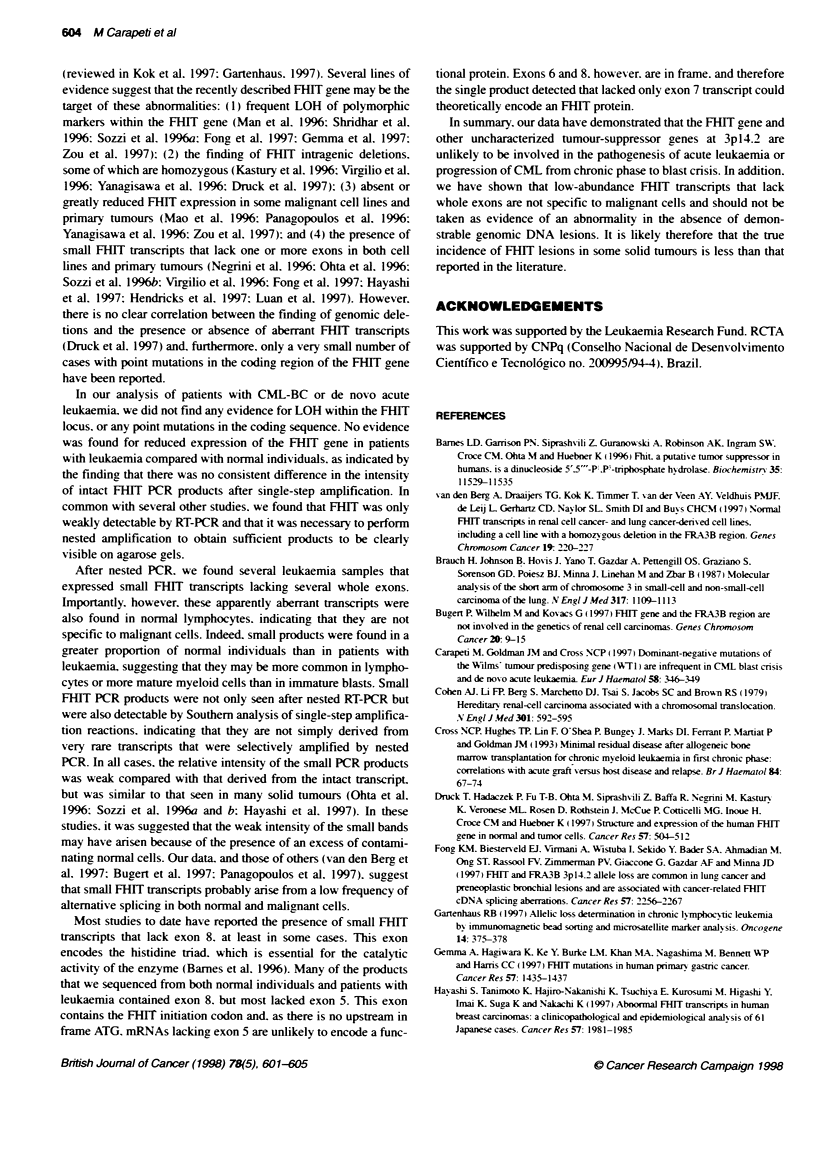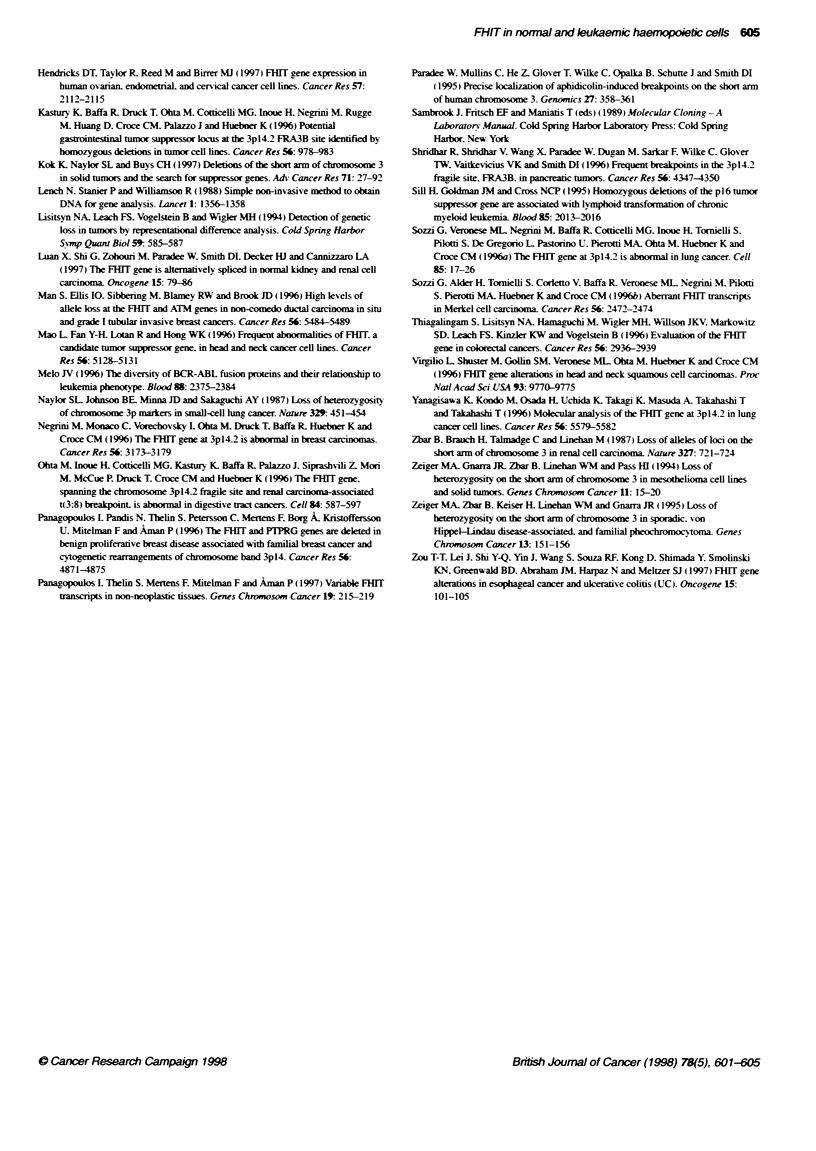# Aberrant transcripts of the FHIT gene are expressed in normal and leukaemic haemopoietic cells.

**DOI:** 10.1038/bjc.1998.547

**Published:** 1998-09

**Authors:** M. Carapeti, R. C. Aguiar, H. Sill, J. M. Goldman, N. C. Cross

**Affiliations:** Department of Haematology, Imperial College School of Medicine, Hammersmith Hospital, London, UK.

## Abstract

**Images:**


British Journal of Cancer (1998) 78(5). 601–605
© 1998 Cancer Research Campaign

### Aberrant transcripts of the FHIT gene are expressed in normal and leukaemic haemopoietic cells

**M Carapeti[1], RCT Aguiar[2], H Sill[3], JM Goldman[1] and NCP Cross[1]**

[1]Department of Haematology. Imperial College School of Medicine. Hammersmith Hospital. Du Cane Road. London W12 0NN. UK; [2]Division of Hematologic Malignancies. Dana Farber Cancer Institute. 44 Binney Street. Boston. MA 02115. USA; [3]Division of Haematology. Department of Internal Medicine. Karl-Franzens University. Auenbruggerplatz 15. A-8036 Graz. Austria

**Summary** Deletions and apparent transcriptional abnormalities of the FHIT gene at 3p14.2 have recently been reported in a wide variety of solid tumours. To determine whether lesions of this gene also occur in leukaemia, we have analysed a total of 97 patients (chronic myeloid leukaemia, CML, in chronic phase or blast crisis, $n = 71$; de novo acute leukaemia, $n = 26$) and 16 normal individuals. Intact FHIT transcripts from all cases were amplified using RT-PCR. Inaddition,smaller size bands that were less intense than the full-length products were amplified from several samples from patients with leukaemia and also from normal leucocytes. Sequencing of the small products revealed that they were derived from FHIT transcripts lacking whole exons. Using single-strand conformation polymorphism analysis, no mutations in the coding sequence were detected in any patient. Furthermore, loss of heterozygosity was not seen in any of 36 informative patients at D3S1300 or D3S1481, markers located within the FHIT locus. We conclude that the FHIT gene and other uncharacterized tumour-suppressor genes at 3p14.2 are unlikely to be involved in the pathogenesis of acute leukaemia or progression of CML from chronic phase to blast crisis. Moreover, low-abundance FHIT transcripts that lack whole exons are not specific to malignant cells and should not be taken as evidence of an abnormality in the absence of demonstrable genomic DNA lesions.

**Keywords:** FHIT; FRA3B; loss of heterozygosity; leukaemia; tumour-suppressor gene; 3p14.2

Allelic deletions of at least three discrete regions of the short arm of chromosome 3 have been found in diverse human cancers and may indicate the sites of novel tumour-suppressor genes (Brauch et al. 1987; Naylor et al. 1987; Zbar et al. 1987; Lisitsyn et al. 1994; Zeiger et al. 1994. 1995; Kastury et al. 1996). One such site is at 3p14.2. a location that was first highlighted by a reciprocal chromosomal translocation. t(3;8)(p14.2;q24). in a family with hereditary renal cell carcinoma (RCC; Cohen et al. 1979). This site also coincides with the most common aphidicolin-inducible fragile site in humans. FRA3B (Paradee et al. 1995). Recently. a novel gene. FHIT (fragile histidine triad). that maps to chromosome 3p14.2 has been implicated as the target of these abnormalities (Ohta et al. 1996). The human FHIT protein has dinucleoside 5′,5‴-$P^1$,$P^3$-triphosphate ($Ap_3A$) hydrolase activity and belongs to a family of proteins that are highly conserved in evolution. The precise cellular function of these proteins is unclear but they may be involved in the regulation of DNA replication and signalling stress responses (Barnes et al. 1996). The FHIT locus consists of ten exons distributed over at least 500 kb and is transcribed as a 1.1 kb mRNA (Figure 1; Ohta et al. 1996).

Absence of FHIT mRNA has been reported in some head and neck squamous cell carcinoma (HNSCC) and oesophageal cell lines (Mao et al. 1996; Zou et al. 1997). but by far the most commonly reported FHIT abnormalities in primary tumour samples are the presence of small aberrant transcripts that lack two or more exons. Such apparent aberrations have been found using reverse transcription polymerase chain reaction (RT-PCR) in a large proportion of cases of oesophageal and gastrointestinal tumours (Ohta et al. 1996). small-cell and non-small-cell lung carcinomas (Sozzi et al. 1996*a*). HNSCC (Mao et al. 1996; Virgilio et al. 1996). Merkel cell carcinomas (Sozzi et al. 1996*b*) and breast carcinomas (Negrini et al. 1996). However. no small transcripts were detected in colorectal tumours (Thiagalingam et al. 1996).

As FHIT is widely expressed. it may also be involved in the pathogenesis of other malignancies. The aim of this study was to determine whether abnormalities of this gene are found in acute leukaemia. including blast crisis of chronic myeloid leukaemia.

*Received 4 November 1997*
*Revised 27 January 1998*
*Accepted 28 January 1998*

*Correspondence to:* NCP Cross

### MATERIALS AND METHODS

### Patient and control samples

Expression of the FHIT gene was evaluated in 119 peripheral blood (PB) or bone marrow (BM) samples obtained from a total of 97 individuals with leukaemia and 16 normal healthy volunteers. Leukaemia samples were from patients with chronic myeloid leukaemia in blast crisis (CML-BC. $n = 51$). acute myeloid leukaemia (AML. $n = 15$). acute lymphoblastic leukaemia (ALL. $n = 8$). unclassified acute leukaemia ($n = 3$) and CML in chronic phase (CML-CP. $n = 26$). For six individuals with CML. both chronic phase and blast crisis samples were analysed. All acute leukaemia and CML-BC samples contained at least 75% blast cells; all CML-CP samples were derived from patients with 100% Philadelphia chromosome-positive bone marrow metaphases.

For loss of heterozygosity (LOH) analysis. a total of 44 paired DNA samples from patients with CML were investigated. DNA from patients in blast crisis was compared with chronic-phase DNA ($n = 28$). buccal epithelial cell DNA ($n = 12$) or both ($n = 4$).

**Figure 1** A schematic representation of the FHIT gene in 3p14.2 showing the position of the internal microsatellite markers (D3S1300 and D3S1481), FRA3B fragile site, t(3;8) translocation breakpoint associated with RCC, and the position of FHIT exons. Black and white boxes represent coding and non-coding exons respectively (E1–E10)

**Figure 2** Agarose gel showing nested PCR products of FHIT with normal sized transcripts (N) as well as aberrant transcripts (A) of various sizes. Lanes 1–8 are from patients with CML in chronic phase or blast crisis; lanes 9–15 are from normal bone marrow or peripheral blood leucocytes

### RT-PCR

Mononuclear cells were isolated using density gradient centrifugation, and total cellular RNA was prepared by lysis in guanidine thiocyanate and caesium chloride ultracentrifugation as described (Sambrook et al, 1989). Two to ten micrograms of total RNA (equivalent to approximately $10^7$ cells) was used for first-strand cDNA synthesis using random hexamer primers. The integrity of all cDNAs was verified by single-step amplification of the normal ABL gene as described (Cross et al, 1993). To test for normal and abnormal FHIT transcripts, a two-step nested PCR was used to amplify the entire coding region using primers (5U2, 5U1, 3D2 and 3D1) and conditions essentially identical to those described previously (Ohta et al, 1996).

### Sequencing

FHIT PCR products were gel purified and directly sequenced in both orientations by thermal cycling with fluorescent dye terminators using primers FHIT-F (5′-TACATCCAGACGGTGGA-3′) and FHIT-R (5′-GGTCTTCAAACTGGTTG-3′), which were internal to the second-step primers 5U1 and 3D1. Reactions were analysed on an automated sequencer (model ABI 373A, Applied Biosystems, Foster City, CA, USA)

### LOH analysis

DNA was extracted as previously described (Lench et al, 1988). PCR amplification was performed for two polymorphic markers, D3S1300 and D3S1481, which are located within the FHIT gene (Kastury et al, 1996; Figure 1). Oligonucleotide sequences were obtained from the Genome Data Base (Johns Hopkins University, Baltimore, MD, USA). Fifty nanograms of genomic DNA from paired samples was amplified for 33 cycles with denaturation at 96°C, annealing at 55°C for D3S1300 and 46°C for D3S1481, followed by extension at 72°C. Products were labelled by reamplification for three cycles with the addition of 2 μCi [α-$^{32}$P]dCTP and fractionated on 6% non-denaturing polyacrylamide gels.

**Table 1** Aberrant transcripts in leukaemic and normal haemopoietic cells

| Sample type (N/L) | Aberrant transcript | Exon 10 11-bp Deletion |
|---|---|---|
| L1, L2, L3 | Deletion exons 4–6 | + |
| L4 | Deletion exons 4–6 | – |
| L5 | Deletion exons 4–7 | + |
| L6 | Deletion exons 4–8 | + |
| L7 | Deletion exons 5–7 | + |
| L8 | Deletion exons 7[a] | + |
| N1, N2 | Deletion exons 4–6 | + |
| N3, N4, N5 | Deletion exons 5–6 | + |
| N6 | Deletion exons 5–8 | + |

[a]Apparently normal size on agarose gel. L, leukaemia; N, normal haemopoietic samples.

### SSCP analysis

Detection of point mutations using single-strand conformation polymorphism analysis (SSCP) was performed as described previously (Carapeti et al, 1997). Briefly, 2 μl of FHIT PCR products encompassing the entire coding region was labelled by reamplification for three cycles, including 2 μCi [α-$^{32}$P]dCTP per reaction and lowering the cold dCTP final concentration to 3 μM. Labelled products were digested with Bsp1286I to yield fragments of 275 bp, 232 bp and 199 bp for intact FHIT transcripts. Digests were diluted tenfold in 0.1% sodium dodecyl sulphate (SDS), 10 mM EDTA, denatured by boiling for 5 min and electrophoresed on 6% non-denaturing polyacrylamide gels at room temperature with or without 8% glycerol.

### RESULTS

### RT-PCR

After single-step PCR, products of the expected size for the intact FHIT mRNA were amplified from most samples, but the intensity of the bands was weak. No consistent differences in intensity were apparent between samples from normal individuals and patients with leukaemia, including those who had >95% blast cells. After nested PCR, intact FHIT transcripts were amplified from all 103

© Cancer Research Campaign 1998

**Figure 3** Sequence chromatograms showing (**A**) deletion of exons 4–7 in a patient with leukaemia, (**B**) deletion of exons 5–8 in a normal individual and (**C**) an 11-bp alternative splice in a normal individual patient samples and the 16 normal individuals. In addition, one or more smaller size bands that were less intense than the full-length products (Figure 2) were amplified from several samples from patients with leukaemia [CML-BC, $n$ = 13 (25%); CML-CP, $n$ = 16 (62%); AML, $n$ = 1 (7%)] and also from normal individuals [$n$ = 13 (76%)]. Southern analysis indicated that the same small FHIT products detected by nested PCR were also present after single-step PCR. No difference in the pattern of amplification products was seen between CML-CP and -BC samples from the same individual for the six cases tested (not shown).

### Sequence analysis

Most apparently normal-sized PCR transcripts, as well as some aberrant splice products, had deletions of the first 11 bp of exon 10 (Figure 3). These deletions are downstream of the TGA stop codon and have been reported previously to result from alternative splicing (Mao et al, 1996; Yanagisawa et al, 1996). Analysis of 14 representative small PCR products derived from both leukaemia samples and normal individuals revealed a range of FHIT transcripts that lacked different combinations of whole exons (Table 1, Figure 3). Fusion products were detected with the following exons spliced together: exons 3–7 ($n$ = 6), exons 3–8 ($n$ = 1), exons 3–9 ($n$ = 1), exons 4–8 ($n$ = 1), exons 6–8 ($n$ = 1), exons 4–7 ($n$ = 3) and exons 4–9 ($n$ = 1).

### LOH analysis

Expected-size PCR products of 241 bp for D3S1300 and 104 bp for D3S1481 were amplified in all cases tested. Of the 44 paired samples amplified for D3S1300, 29 (66%) were informative. For D3S1481, 21 (78%) of the 27 cases tested were informative. Overall, 36 (82%) of the 44 paired samples were informative for at least one of the loci. LOH was not detected in any of the informative cases at either locus.

### SSCP

SSCP analysis was performed for 67 samples (CML-BC, $n$ = 45; CML-CP, $n$ = 3; AML, $n$ = 14; ALL, $n$ = 2; AL, $n$ = 3). In addition to the 11-bp deletion in the 3′ untranslated region (UTR) described above, band shifts for one fragment were noted for four samples (6%). Sequence analysis revealed two previously reported silent polymorphisms: a C to T change at codon 88 (alanine, GCC → GCT) and a T to C change at codon 98 (histidine, CAT → CAC) (Mao et al, 1996; Thiagalingam et al, 1996; Yanagisawa et al, 1996). No other changes were found.

### DISCUSSION

CML is a clonal myeloproliferative disorder that usually presents in chronic phase but eventually progresses to an acute leukaemia (blast crisis) in almost all patients. Although the BCR-ABL fusion gene can be detected in 90–95% of cases and plays a central role in the pathogenesis of chronic phase CML (Melo, 1996), little is known about the molecular events responsible for the transformation from chronic phase to blast crisis (Sill et al, 1995). Similarly, a complete description of the molecular pathogenesis of de novo acute leukaemia is lacking.

Allelic deletions at 3p14.2 have been reported in a wide variety of solid tumours and also in chronic lymphocytic leukaemia

© Cancer Research Campaign 1998

(reviewed in Kok et al. 1997; Gartenhaus, 1997). Several lines of evidence suggest that the recently described FHIT gene may be the target of these abnormalities: (1) frequent LOH of polymorphic markers within the FHIT gene (Man et al. 1996; Shridhar et al. 1996; Sozzi et al. 1996*a*; Fong et al. 1997; Gemma et al. 1997; Zou et al. 1997); (2) the finding of FHIT intragenic deletions, some of which are homozygous (Kastury et al. 1996; Virgilio et al. 1996; Yanagisawa et al. 1996; Druck et al. 1997); (3) absent or greatly reduced FHIT expression in some malignant cell lines and primary tumours (Mao et al. 1996; Panagopoulos et al. 1996; Yanagisawa et al. 1996; Zou et al. 1997); and (4) the presence of small FHIT transcripts that lack one or more exons in both cell lines and primary tumours (Negrini et al. 1996; Ohta et al. 1996; Sozzi et al. 1996*b*; Virgilio et al. 1996; Fong et al. 1997; Hayashi et al. 1997; Hendricks et al. 1997; Luan et al. 1997). However, there is no clear correlation between the finding of genomic deletions and the presence or absence of aberrant FHIT transcripts (Druck et al. 1997) and, furthermore, only a very small number of cases with point mutations in the coding region of the FHIT gene have been reported.

In our analysis of patients with CML-BC or de novo acute leukaemia, we did not find any evidence for LOH within the FHIT locus, or any point mutations in the coding sequence. No evidence was found for reduced expression of the FHIT gene in patients with leukaemia compared with normal individuals, as indicated by the finding that there was no consistent difference in the intensity of intact FHIT PCR products after single-step amplification. In common with several other studies, we found that FHIT was only weakly detectable by RT-PCR and that it was necessary to perform nested amplification to obtain sufficient products to be clearly visible on agarose gels.

After nested PCR, we found several leukaemia samples that expressed small FHIT transcripts lacking several whole exons. Importantly, however, these apparently aberrant transcripts were also found in normal lymphocytes, indicating that they are not specific to malignant cells. Indeed, small products were found in a greater proportion of normal individuals than in patients with leukaemia, suggesting that they may be more common in lymphocytes or more mature myeloid cells than in immature blasts. Small FHIT PCR products were not only seen after nested RT-PCR but were also detectable by Southern analysis of single-step amplification reactions, indicating that they are not simply derived from very rare transcripts that were selectively amplified by nested PCR. In all cases, the relative intensity of the small PCR products was weak compared with that derived from the intact transcript, but was similar to that seen in many solid tumours (Ohta et al. 1996; Sozzi et al. 1996*a* and *b*; Hayashi et al. 1997). In these studies, it was suggested that the weak intensity of the small bands may have arisen because of the presence of an excess of contaminating normal cells. Our data, and those of others (van den Berg et al. 1997; Bugert et al. 1997; Panagopoulos et al. 1997), suggest that small FHIT transcripts probably arise from a low frequency of alternative splicing in both normal and malignant cells.

Most studies to date have reported the presence of small FHIT transcripts that lack exon 8, at least in some cases. This exon encodes the histidine triad, which is essential for the catalytic activity of the enzyme (Barnes et al. 1996). Many of the products that we sequenced from both normal individuals and patients with leukaemia contained exon 8, but most lacked exon 5. This exon contains the FHIT initiation codon and, as there is no upstream in frame ATG, mRNAs lacking exon 5 are unlikely to encode a functional protein. Exons 6 and 8, however, are in frame, and therefore the single product detected that lacked only exon 7 transcript could theoretically encode an FHIT protein.

In summary, our data have demonstrated that the FHIT gene and other uncharacterized tumour-suppressor genes at 3p14.2 are unlikely to be involved in the pathogenesis of acute leukaemia or progression of CML from chronic phase to blast crisis. In addition, we have shown that low-abundance FHIT transcripts that lack whole exons are not specific to malignant cells and should not be taken as evidence of an abnormality in the absence of demonstrable genomic DNA lesions. It is likely therefore that the true incidence of FHIT lesions in some solid tumours is less than that reported in the literature.

### ACKNOWLEDGEMENTS

This work was supported by the Leukaemia Research Fund. RCTA was supported by CNPq (Conselho Nacional de Desenvolvimento Científico e Tecnológico no. 200995/94-4), Brazil.

### REFERENCES

Barnes LD, Garrison PN, Siprashvili Z, Guranowski A, Robinson AK, Ingram SW, Croce CM, Ohta M and Huebner K (1996) Fhit, a putative tumor suppressor in humans, is a dinucleoside 5',5'''-P¹,P³-triphosphate hydrolase. *Biochemistry* **35**: 11529–11535

van den Berg A, Draaijers TG, Kok K, Timmer T, van der Veen AY, Veldhuis PMJF, de Leij L, Gerhartz CD, Naylor SL, Smith DI and Buys CHCM (1997) Normal FHIT transcripts in renal cell cancer- and lung cancer-derived cell lines, including a cell line with a homozygous deletion in the FRA3B region. *Genes Chromosom Cancer* **19**: 220–227

Brauch H, Johnson B, Hovis J, Yano T, Gazdar A, Pettengill OS, Graziano S, Sorenson GD, Poiesz BJ, Minna J, Linehan M and Zbar B (1987) Molecular analysis of the short arm of chromosome 3 in small-cell and non-small-cell carcinoma of the lung. *N Engl J Med* **317**: 1109–1113

Bugert P, Wilhelm M and Kovacs G (1997) FHIT gene and the FRA3B region are not involved in the genetics of renal cell carcinomas. *Genes Chromosom Cancer* **20**: 9–15

Carapeti M, Goldman JM and Cross NCP (1997) Dominant-negative mutations of the Wilms' tumour predisposing gene (WT1) are infrequent in CML blast crisis and de novo acute leukaemia. *Eur J Haematol* **58**: 346–349

Cohen AJ, Li FP, Berg S, Marchetto DJ, Tsai S, Jacobs SC and Brown RS (1979) Hereditary renal-cell carcinoma associated with a chromosomal translocation. *N Engl J Med* **301**: 592–595

Cross NCP, Hughes TP, Lin F, O'Shea P, Bungey J, Marks DI, Ferrant P, Martiat P and Goldman JM (1993) Minimal residual disease after allogeneic bone marrow transplantation for chronic myeloid leukaemia in first chronic phase: correlations with acute graft versus host disease and relapse. *Br J Haematol* **84**: 67–74

Druck T, Hadaczek P, Fu T-B, Ohta M, Siprashvili Z, Baffa R, Negrini M, Kastury K, Veronese ML, Rosen D, Rothstein J, McCue P, Cotticelli MG, Inoue H, Croce CM and Huebner K (1997) Structure and expression of the human FHIT gene in normal and tumor cells. *Cancer Res* **57**: 504–512

Fong KM, Biesterveld EJ, Virmani A, Wistuba I, Sekido Y, Bader SA, Ahmadian M, Ong ST, Rassool FV, Zimmerman PV, Giaccone G, Gazdar AF and Minna JD (1997) FHIT and FRA3B 3p14.2 allele loss are common in lung cancer and preneoplastic bronchial lesions and are associated with cancer-related FHIT cDNA splicing aberrations. *Cancer Res* **57**: 2256–2267

Gartenhaus RB (1997) Allelic loss determination in chronic lymphocytic leukemia by immunomagnetic bead sorting and microsatellite marker analysis. *Oncogene* **14**: 375–378

Gemma A, Hagiwara K, Ke Y, Burke LM, Khan MA, Nagashima M, Bennett WP and Harris CC (1997) FHIT mutations in human primary gastric cancer. *Cancer Res* **57**: 1435–1437

Hayashi S, Tanimoto K, Hajiro-Nakanishi K, Tsuchiya E, Kurosumi M, Higashi Y, Imai K, Suga K and Nakachi K (1997) Abnormal FHIT transcripts in human breast carcinomas: a clinicopathological and epidemiological analysis of 61 Japanese cases. *Cancer Res* **57**: 1981–1985

© Cancer Research Campaign 1998

Hendricks DT, Taylor R, Reed M and Birrer MJ (1997) FHIT gene expression in human ovarian, endometrial, and cervical cancer cell lines. *Cancer Res* **57**: 2112–2115

Kastury K, Baffa R, Druck T, Ohta M, Cotticelli MG, Inoue H, Negrini M, Rugge M, Huang D, Croce CM, Palazzo J and Huebner K (1996) Potential gastrointestinal tumor suppressor locus at the 3p14.2 FRA3B site identified by homozygous deletions in tumor cell lines. *Cancer Res* **56**: 978–983

Kok K, Naylor SL and Buys CH (1997) Deletions of the short arm of chromosome 3 in solid tumors and the search for suppressor genes. *Adv Cancer Res* **71**: 27–92

Lench N, Stanier P and Williamson R (1988) Simple non-invasive method to obtain DNA for gene analysis. *Lancet* **1**: 1356–1358

Lisitsyn NA, Leach FS, Vogelstein B and Wigler MH (1994) Detection of genetic loss in tumors by representational difference analysis. *Cold Spring Harbor Symp Quant Biol* **59**: 585–587

Luan X, Shi G, Zohouri M, Paradee W, Smith DI, Decker HJ and Cannizzaro LA (1997) The FHIT gene is alternatively spliced in normal kidney and renal cell carcinoma. *Oncogene* **15**: 79–86

Man S, Ellis IO, Sibbering M, Blamey RW and Brook JD (1996) High levels of allele loss at the FHIT and ATM genes in non-comedo ductal carcinoma in situ and grade I tubular invasive breast cancers. *Cancer Res* **56**: 5484–5489

Mao L, Fan Y-H, Lotan R and Hong WK (1996) Frequent abnormalities of FHIT, a candidate tumor suppressor gene, in head and neck cancer cell lines. *Cancer Res* **56**: 5128–5131

Melo JV (1996) The diversity of BCR-ABL fusion proteins and their relationship to leukemia phenotype. *Blood* **88**: 2375–2384

Naylor SL, Johnson BE, Minna JD and Sakaguchi AY (1987) Loss of heterozygosity of chromosome 3p markers in small-cell lung cancer. *Nature* **329**: 451–454

Negrini M, Monaco C, Vorechovsky I, Ohta M, Druck T, Baffa R, Huebner K and Croce CM (1996) The FHIT gene at 3p14.2 is abnormal in breast carcinomas. *Cancer Res* **56**: 3173–3179

Ohta M, Inoue H, Cotticelli MG, Kastury K, Baffa R, Palazzo J, Siprashvili Z, Mori M, McCue P, Druck T, Croce CM and Huebner K (1996) The FHIT gene, spanning the chromosome 3p14.2 fragile site and renal carcinoma-associated t(3:8) breakpoint, is abnormal in digestive tract cancers. *Cell* **84**: 587–597

Panagopoulos I, Pandis N, Thelin S, Petersson C, Mertens F, Borg Å, Kristoffersson U, Mitelman F and Åman P (1996) The FHIT and PTPRG genes are deleted in benign proliferative breast disease associated with familial breast cancer and cytogenetic rearrangements of chromosome band 3p14. *Cancer Res* **56**: 4871–4875

Panagopoulos I, Thelin S, Mertens F, Mitelman F and Åman P (1997) Variable FHIT transcripts in non-neoplastic tissues. *Genes Chromosom Cancer* **19**: 215–219

Paradee W, Mullins C, He Z, Glover T, Wilke C, Opalka B, Schutte J and Smith DI (1995) Precise localization of aphidicolin-induced breakpoints on the short arm of human chromosome 3. *Genomics* **27**: 358–361

Sambrook J, Fritsch EF and Maniatis T (eds) (1989) *Molecular Cloning – A Laboratory Manual*. Cold Spring Harbor Laboratory Press: Cold Spring Harbor, New York

Shridhar R, Shridhar V, Wang X, Paradee W, Dugan M, Sarkar F, Wilke C, Glover TW, Vaitkevicius VK and Smith DI (1996) Frequent breakpoints in the 3p14.2 fragile site, FRA3B, in pancreatic tumors. *Cancer Res* **56**: 4347–4350

Sill H, Goldman JM and Cross NCP (1995) Homozygous deletions of the p16 tumor suppressor gene are associated with lymphoid transformation of chronic myeloid leukemia. *Blood* **85**: 2013–2016

Sozzi G, Veronese ML, Negrini M, Baffa R, Cotticelli MG, Inoue H, Tornielli S, Pilotti S, De Gregorio L, Pastorino U, Pierotti MA, Ohta M, Huebner K and Croce CM (1996*a*) The FHIT gene at 3p14.2 is abnormal in lung cancer. *Cell* **85**: 17–26

Sozzi G, Alder H, Tornielli S, Corletto V, Baffa R, Veronese ML, Negrini M, Pilotti S, Pierotti MA, Huebner K and Croce CM (1996*b*) Aberrant FHIT transcripts in Merkel cell carcinoma. *Cancer Res* **56**: 2472–2474

Thiagalingam S, Lisitsyn NA, Hamaguchi M, Wigler MH, Willson JKV, Markowitz SD, Leach FS, Kinzler KW and Vogelstein B (1996) Evaluation of the FHIT gene in colorectal cancers. *Cancer Res* **56**: 2936–2939

Virgilio L, Shuster M, Gollin SM, Veronese ML, Ohta M, Huebner K and Croce CM (1996) FHIT gene alterations in head and neck squamous cell carcinomas. *Proc Natl Acad Sci USA* **93**: 9770–9775

Yanagisawa K, Kondo M, Osada H, Uchida K, Takagi K, Masuda A, Takahashi T and Takahashi T (1996) Molecular analysis of the FHIT gene at 3p14.2 in lung cancer cell lines. *Cancer Res* **56**: 5579–5582

Zbar B, Brauch H, Talmadge C and Linehan M (1987) Loss of alleles of loci on the short arm of chromosome 3 in renal cell carcinoma. *Nature* **327**: 721–724

Zeiger MA, Gnarra JR, Zbar B, Linehan WM and Pass HI (1994) Loss of heterozygosity on the short arm of chromosome 3 in mesothelioma cell lines and solid tumors. *Genes Chromosom Cancer* **11**: 15–20

Zeiger MA, Zbar B, Keiser H, Linehan WM and Gnarra JR (1995) Loss of heterozygosity on the short arm of chromosome 3 in sporadic, von Hippel–Lindau disease-associated, and familial pheochromocytoma. *Genes Chromosom Cancer* **13**: 151–156

Zou T-T, Lei J, Shi Y-Q, Yin J, Wang S, Souza RF, Kong D, Shimada Y, Smolinski KN, Greenwald BD, Abraham JM, Harpaz N and Meltzer SJ (1997) FHIT gene alterations in esophageal cancer and ulcerative colitis (UC). *Oncogene* **15**: 101–105

© Cancer Research Campaign 1998